# Glucose variability and mode of anaesthesia in major noncardiac surgery (GlucoVITAL): study protocol for a randomised controlled trial

**DOI:** 10.1016/j.bjao.2025.100419

**Published:** 2025-05-24

**Authors:** Henrike Janssen, Priyanthi Dias, Louise Hiller, Russell Hewson, Rupert M. Pearse, Nick S. Oliver, Shaman Jhanji, Gareth L. Ackland, GlucoVITAL Trial Investigators, Ashok Sundar, Islam Abousharkh, Priyanthi Dias, Jai Varale, Joyce Yeung, Rupert Pearse, Benjamin Shelley, Louise Hiller, Kamran Khan, Peter Jacob, James Noblet, Monica Jefford, Toby Reynolds, Ana Gutierrez del Arroyo, Abeer Samman, Saja Alharbi, Sanjali Ahuja, Helen T. Neo Phatsimo, Keagan Lewis Witts, Muhammad Abdullah Pirwani, Su Yen Lee, Madhura Sivakumar, Divyani Priyavadan Bhudia, Marina Lily Chan, Emmanuella Anobah Adu, Marta Korbonits, Sian Henson, Onika Ottley, Mareena Joseph, Augustine Miguel Saavedra, Ana Santos, Eleanor Reeves, Joanne Gresty, Celinamma Maliaykal, Karthik Iyer, Lesley Horton, Helen Taylor, Ashok Nair, Catriona Frankling, Chloe Uffendell, Caroline Thomas, Catherine Moriarty, Beverley Jackson, Daniel Ruane, Reyhaneh Sadegh Zadeh, Ethel Black, Karmina Claros, Nick Perry, Vasiliki Manou-Stathopoulou, Ravishankar Rao Baikady, Holly Townsend-Thorn, Nicole Whitehead, Kate Tatham, Sophie Uren, Richard Gordon-Williams, Don Milliken, Jaishel Patel, Maxime Cordon-Goudreau, Edward Watson, Tom Craig, Francesca Mazzola, Richard Snooks, Sameer Ganatra

**Affiliations:** 1 Faculty of Medicine and Dentistry, William Harvey Research Institute, Queen Mary University of London, London, UK; 2 Warwick Clinical Trials Unit, University of Warwick, Coventry, UK; 3 Department of Metabolism, Digestion and Reproduction, Faculty of Medicine, Imperial College London, London, UK; 4 Department of Anaesthesia, Perioperative Medicine and Critical Care, Royal Marsden Hospital, London, UK

**Keywords:** continuous glucose monitoring, glucose, infections, myocardial injury, postoperative complications

## Abstract

**Background:**

Hyperglycaemia after noncardiac surgery occurs commonly and is associated with complications. The choice of maintenance anaesthesia may promote hyperglycaemia and increase glucose variability, both of which exacerbate inflammation and organ dysfunction. We hypothesise that total intravenous anaesthesia reduces glucose variability, particularly in individuals with insulin resistance or diabetes mellitus, and hence may reduce postoperative complications.

**Methods:**

This multicentre, randomised controlled parallel group trial will recruit 450 participants ≥50 yr undergoing elective noncardiac surgery. Participants will be randomly allocated in a 1:1 ratio (with minimisation) to receive either total intravenous anaesthesia or inhalation agents (typically sevoflurane) for maintenance of anaesthesia. The primary outcome is blood glucose, measured at prespecified timepoints (before, immediately after, and the morning after surgery). Continuous glucose monitoring (CGM; Dexcom G7) will commence at induction of anaesthesia for up to 10 days after surgery (or hospital discharge) to establish the reliability and accuracy of CGM compared with blood glucose measurements. Secondary outcomes include days alive and out of hospital within 30 days of surgery and postoperative complications (Clavien–Dindo grade ≥2). Absolute glucose and CGM-derived measures of glucose variability will be compared between participants who sustain, or remain free of, myocardial injury within 24 h of surgery, infectious complications within 30 days of surgery, and vasopressor use persisting >4 h after surgery.

**Conclusions:**

GlucoVITAL will establish whether the mode of anaesthesia may alter glucose control in susceptible individuals and also explore the role of glucose variability in organ injury after noncardiac surgery using CGM.

**Clinical trial registration:**

ISRCTN46862025.

An increasing proportion of individuals undergoing noncardiac surgery have insulin resistance or a clinical diagnosis of diabetes mellitus.^1^ Preoperative fasting hyperglycaemia is associated with an increased risk of adverse postoperative cardiovascular events.^2^ Perioperative stress-induced hyperglycaemia in response to tissue injury is also associated with postoperative complications.^2^^,^^3^ Observational studies in noncardiac surgery suggest that people without diabetes have a substantially higher risk of acquiring serious complications when controlling for the same level of hyperglycaemia as people with established diabetes mellitus.^4^^,^^5^ The type of anaesthesia may, in part, influence the duration and severity of stress hyperglycaemia. Volatile anaesthetics impair insulin signalling, inhibit insulin secretion by opening K_ATP_ channels in β cells of the pancreas,^6^ and induce hepatic insulin resistance rapidly,^7^^,^^8^ which may promote more severe hyperglycaemia compared with TIVA.^9^

The failure to prevent, or even detect, brief hyperglycaemic periods during noncardiac surgery may account for many infections after surgery and additional organ injury, leading to prolonged hospitalisation and poorer long-term outcomes. Brief exposure to higher circulating levels of glucose (∼16 mmol L^−1^) fuels systemic inflammation^10^ and transiently reduces endothelial-dependent vasodilation,^11^ suggesting glucose variability may also contribute to postoperative vasoplegia. In support of these experimental findings, tight blood-glucose control without early parenteral nutrition, comprising ∼51% postoperative patients admitted to ICU, reduced severe renal injury, new use of renal replacement therapy, or both.^12^ However, a detailed examination of the interaction between glucose variability, inflammation, and mode of anaesthesia has not been possible until the advent of continuous glucose monitoring (CGM),^13^ which affords an unprecedented insight into the perioperative stress response.^14^

The purpose of this protocol report is to describe the evolution of GlucoVITAL from being a nested observational cohort study embedded in the multicentre VITAL RCT to a stand-alone RCT, and the translational studies that complement the primary research question.

## Methods

### Study design

The study was initially undertaken as an observational study involving six centres nested within the RCT Volatile vs Total Intravenous Anaesthesia for major non-cardiac surgery (VITAL) trial (ISRCTN: 62903453), which randomly allocated patients to receive TIVA or volatile-based anaesthesia in adults ≥50 yr undergoing elective major noncardiac surgery under general anaesthesia.^15^ After the VITAL RCT achieved its final target enrolment early in April 2024, the funder (National Institute for Healthcare Research Efficacy and Mechanism Evaluation funding stream: NIHR154842) and sponsor (Queen Mary University of London [QMUL]) agreed to continue and complete GlucoVITAL by transitioning the study to become a stand-alone, open-label multicentre randomised trial using the same criteria as VITAL. Ethical permission was granted by the Health Research Authority (London-Fulham Research Ethics Committee; REC reference: 23/PR/0677) to convert GlucoVITAL from an observational study nested within VITAL (granted on August 17, 2023) into a stand-alone RCT on this basis on June 13, 2024. The original and updated trial details were registered prospectively with the UK Clinical Study Registry (ISRCTN 46862025). Authors will have access to information that could identify individual participants during or after data collection.

### Inclusion and exclusion criteria

Patients will be eligible for inclusion if they are aged ≥50 yr, scheduled for elective major noncardiac surgery under general anaesthesia (as per original VITAL inclusion criteria), and provide written informed consent for trial participation. Exclusion criteria will be a known contraindication to either TIVA or inhalation anaesthesia, refusal to participate by the treating clinician, procedures where the patient is not expected to survive for 30 days, previous participation to completion in the VITAL or GlucoVITAL studies, and inability to give informed consent or complete questionnaires.

### Screening and consent

**Figure 1:**
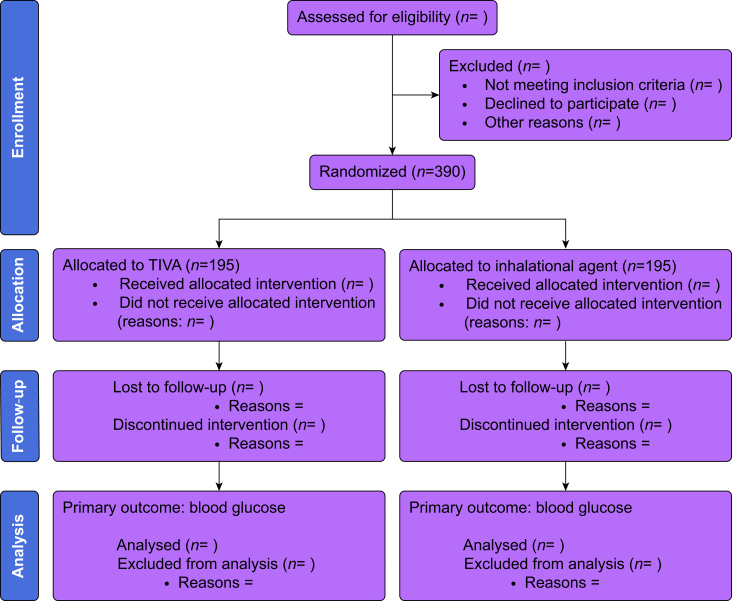
Consolidated Standards of Reporting Trials (CONSORT) summary of analysis for primary outcome.

Potential participants are screened by research staff at the site having been identified from pre-admission clinic lists, operating theatre lists, and by communication with the relevant nursing and medical staff (Fig. 1). Written informed consent is obtained before surgery.

### Randomisation

Participants will be randomly allocated by research staff after enrolment using a web-based portal on a 1:1 basis to receive either TIVA or inhalation agents (typically sevoflurane) for the maintenance of anaesthesia. This computerised procedure uses a minimisation algorithm from the original VITAL protocol to ensure balance in treatment arm allocation across the following four stratification variables:

1. Surgical speciality (musculoskeletal/intra-abdominal/thoracic/vascular/other).
2. Expected duration of surgery (</≥2 h).
3. Cancer/non-cancer surgery.
4. Preoperative frailty, classified as well, vulnerable or frail using the Rockwood Frailty Score.

### Intervention

Anaesthesia is administered by experienced medically qualified anaesthetists and delivered according to local guidelines. All other participant care is conducted as per routine clinical practice.

#### Total intravenous anaesthesia

Participants allocated to the TIVA arm of the trial have their maintenance of anaesthesia performed with intravenous anaesthetic agents as determined by the treating anaesthetist. Maintenance of general anaesthesia is by TIVA only, with analgesic management determined by the treating anaesthetist.

#### Inhalation anaesthesia

Participants allocated to the inhalation arm of the trial receive maintenance of anaesthesia with inhalation anaesthetic agents as determined by the treating anaesthetist. The inhalation agent of choice is sevoflurane in contributing hospitals. The induction of anaesthesia with intravenous agents including propofol is permitted. Analgesic management is also left to the discretion of the treating anaesthetist.

#### Glucose measurements

Blood glucose values and their source (arterial, venous, or capillary) are recorded using calibrated ISO 15197:2013 compliant blood gas analysers.^16^ The majority of measurements in this perioperative setting will be made from arterial blood samples. The prespecified times for blood glucose measurements are the day of surgery before induction of anaesthesia, the end of surgery defined as within 2 h of the participant leaving the operating room, and the day after surgery (10:00 ± 6 h).

CGM will be performed using Dexcom G7 CGM CE-marked sensors (Dexcom, San Diego, CA, USA) sited by research staff in the upper, outer arm after induction of anaesthesia. The participant wears CGM sensors continuously from induction of anaesthesia for up to 10 days after operation (maximum usage duration for each sensor) or hospital discharge, whichever occurs sooner. Participants, clinicians, and research staff are blinded to real-time data from the CGM receiver.

### Outcome assessments

All outcome assessments are masked to treatment allocation until the final analysis.

### Primary outcome

**Figure 2:**
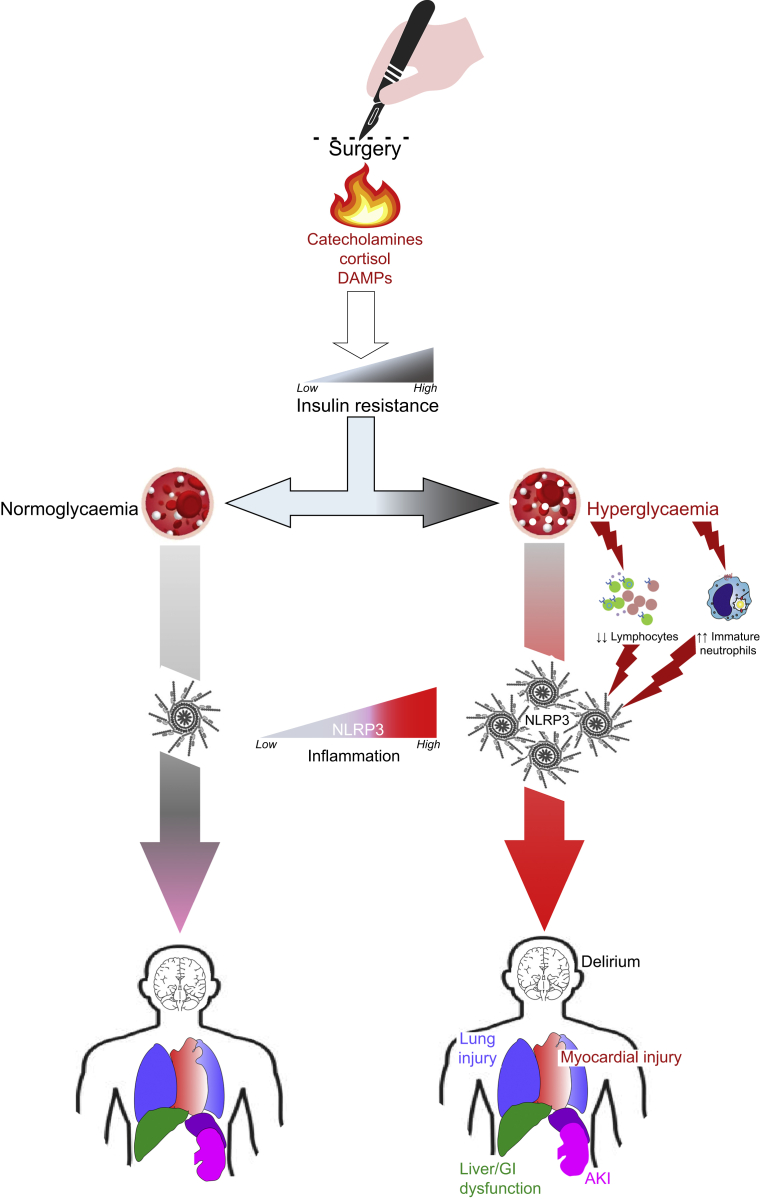
Summary of mechanisms of cell injury mediated by hyperglycaemia on perioperative inflammation and organ dysfunction. Higher glucose concentrations (relative hyperglycaemia) have been demonstrated experimentally to be promoted by acute inflammation, the neurohormonal response to tissue injury, and the physio-pharmacological properties of anaesthesia agents. In turn, hyperglycaemia-induced exacerbation of systemic and local inflammation is driven by NLRP3 (NOD-, LRR-, and pyrin domain-containing protein 3), which is a ubiquitous intracellular sensor that detects a broad range of microbial motifs and endogenous danger signals (DAMPs), resulting in the formation and activation of the NLRP3 inflammasome. Assembly of the NLRP3 inflammasome leads to caspase 1-dependent release of the pro-inflammatory cytokines IL-1β and IL-18. AKI, acute kidney injury; GI, gastrointestinal; IL-1β/18, interleukin 1β/18; DAMP, danger associated molecular patterns.

The primary outcome is the difference in blood glucose at the start and the end of surgery (Fig. 2).

### Secondary outcomes

#### Secondary clinical outcome measures

1. Days alive and at home within 30 days after surgery (DAH30),^17^ where home reflects any place other than hospital. If a participant dies within those 30 days, their value is set to 0. DAH30 captures the development of all-cause complications which prevent participants leaving hospital after surgery.
2. Myocardial injury: serum high-sensitivity troponin-T (Elecsys, Roche Diagnostics, Basel, Switzerland). Myocardial injury is defined by an absolute value of ≥15 ng L^−1^ on day 1 after surgery or an increase of ≥5 ng L^−1^ from the preoperative value on day 1 after surgery when the preoperative value was ≥15 ng L^−1^.^18^
3. Postoperative infection within 30 days after surgery. This is defined as one or more of the following infections meeting Clavien–Dindo grade II criteria (or greater), as successfully used in large multicentre surgical epidemiological studies^19^: superficial surgical site infection, deep surgical site infection, organ space surgical site infection, pneumonia, urinary tract infection, laboratory-confirmed bloodstream infection, and infection of uncertain source.

#### Explanatory mechanistic measures

1. Continuous glucose measurements using CGM up to 10 days after surgery or hospital discharge, whichever is sooner.
2. Paired plasma insulin and glucose measurements before surgery to approximate insulin resistance using the Homeostatic Model Assessment for Insulin Resistance (HOMA-IR).^20^
3. Flow cytometry count of leucocyte subpopulations and metabolic characteristics of leucocytes before surgery, the end of surgery, day 1 after surgery, and at hospital discharge (in selected centres).^21^

### Trial monitoring

An independent trial steering committee comprising senior clinical academics in perioperative medicine and diabetes/metabolism, an independent statistician, and an immunologist approved by the funder will review the trial on a regular basis. The trial steering committee is responsible for recommendations to the trial management group regarding stopping or continuing the trial. If there are adverse events of concern that may involve the use of CGM, a sub-group led by the trial steering committee chair will launch a study review.

### Sample size estimation

For the primary outcome, blood glucose values obtained in the VITAL RCT (before GlucoVITAL was funded) showed that mean blood glucose in 47 participants without diabetes increased by 2.2 mmol/L (standard deviation [sd]: 1.8) by the end of surgery. Assuming 5% two-sided significance and 90% power, with a sd of glucose change by the end of surgery of up to 1.8 mmol L^−1^, at least 390 participants would be required to detect a minimum difference of at least 0.6 mmol L^−1^ in blood glucose between modes of anaesthesia (allowing for nominal 2% dropout). The normal variation in fasting plasma glucose levels is 0.6 mmol L^−1^.^22^

We estimated the number of participants required for the secondary (composite) clinical outcomes based on ∼47% Optimisation of Perioperative Cardiovascular Management to Improve Surgical Outcome (OPTIMISE) trial^23^ and Vascular Events in Noncardiac Surgery Cohort Evaluation (VISION)- United Kingdom^24^ participants >50 yr sustaining myocardial injury within the first 24 h after surgery (∼48%), all-cause infection at any time before hospital discharge (∼20% participants), or both. With 450 participants, we will have a 90% chance of detecting (at 5% two-sided significance) a decrease in this composite outcome from 56% with inhalation *vs* 40% with intravenous anaesthesia (allowing for a 4% dropout). We will also record DAH30, which reflects composite complications after surgery.

### Bias and blinding

It is not possible to blind participants, clinicians, and research staff to the participant's randomised allocation for maintenance of anaesthesia. Participants, clinicians, and research staff will be blinded to CGM and high-sensitivity troponin measurements. Laboratory investigations will be masked to treatment allocation and glucose measurements. During the trial, the trial management group and the trial steering committee will not see outcome results broken down by treatment allocation.

### Data collection

Electronic health records will be screened for postoperative complications. CGM data are exported to a trial-specific platform (Dexcom Clarity: https://clarity.dexcom.eu/). Whole blood samples obtained at the three prespecified timepoints are prepared as plasma samples stored at −80°C for batch analysis. These data will be collected by a member of the research team not involved with administering the interventions, and analysed by an independent member of the research team—both will be masked to the treatment allocations. Participants will be contacted by telephone 30 days after surgery by site research staff to screen for hospital readmission and any postoperative complications that are classed by Clavien–Dindo Severity Grade.

### Data management

Personal data collected during the trial will be handled and stored in accordance with the 2018 Data Protection Act. Participants will be identified using their unique trial number only, and no data which identify participants by name will be shared with nor held at the site of the chief investigator (CI). All data will be entered electronically on a custom-designed database. Clinical data will be collected during the hospital stay up to 30 days after allocation. The case report form is available to each site electronically. On all trial-specific documents, other than the signed consent form, the participant will be referred to using their unique, trial-specific number. Signed consent forms will be retained at the recruiting site and will not be shared with the site of the CI.

### Statistical analysis

A Consolidated Standards of Reporting Trials (CONSORT) summary including a flow diagram will be produced to report eligibility, exclusion before randomisation, allocation to each randomisation arm, and follow-up at prespecified timepoints.^25^ Detailed statistical analysis plans will be available online after approval by the CI and an independent statistician before database lockdown. All statistical analyses will be undertaken on an intention-to-treat basis to preserve randomisation, avoid bias from exclusions, and preserve statistical power. Hence, all participants enrolled into the study, regardless of whether they received their randomised intervention, will be analysed according to their randomised group using data collected up to their 30-day time-point, or the last time-point before their withdrawal or loss to follow-up before this. Participants not receiving surgery or withdrawing consent for follow-up before surgery will not be included in relevant denominators.

For the primary outcome, blood glucose measurements from the start of surgery until the end of surgery will be compared across randomised treatment arms using independent samples *t*-tests or Wilcoxon rank sum tests depending on the distribution of the data. The secondary clinical outcomes (incidence of myocardial injury, all-cause infection, or both) will be assessed across trial arms using a chi-squared test. The continuous variables measuring mechanistic outcomes of glucose variability profiles will be compared across trial arms using repeated-measures analyses, a statistically efficient approach that allows all of the follow-up data collated during the study to be used. Prespecified sub-group analyses will be undertaken using appropriate modelling techniques. These will be determined after examination of the distributions of the collected data. These exploratory sub-group analyses will have lower power than the main whole-trial analysis but are hypothesis-generating and results will be scrutinised graphically using forest plots.

### Trial management and data monitoring

The sponsor organisation is QMUL. Daily trial management is coordinated by a trial management group consisting of the CI and their support staff. A trial steering committee oversees the trial, including assessing the safety of the intervention, reviewing relevant new external evidence, and monitoring the overall conduct of the trial. This committee consists of an independent clinical trialist, lay representative, and an independent chair.

### Adverse events

Prespecified adverse events directly related to CGM sensors will be recorded, including local skin irritation, bruising, haematoma, and infection.

### Auditing

The sponsor will have oversight of the trial conduct. The trial team will ensure compliance with the requirements of Good Clinical Practice including data quality control and safety reporting.

### Dissemination plans

There are six prespecified separate analyses that will be reported from data obtained in the GlucoVITAL trial. Statistical analysis plans will be publicly available at https://www.qmul.ac.uk/ccpmg/sops--saps/statistical-analysis-plans-saps.

1. Accuracy of CGM sensor data, compared with blood glucose values, when CGM is commenced in the acute perioperative period. Although there are previous small reports using CGM in the noncardiac surgical setting,^8^ this analysis is an essential first step to use CGM-derived variability data for our planned subsequent mechanistic work in GlucoVITAL.
2. The impact of mode of maintenance anaesthesia on perioperative glycaemic control and glycaemic variability (Fig. 3).

**Figure 3:**
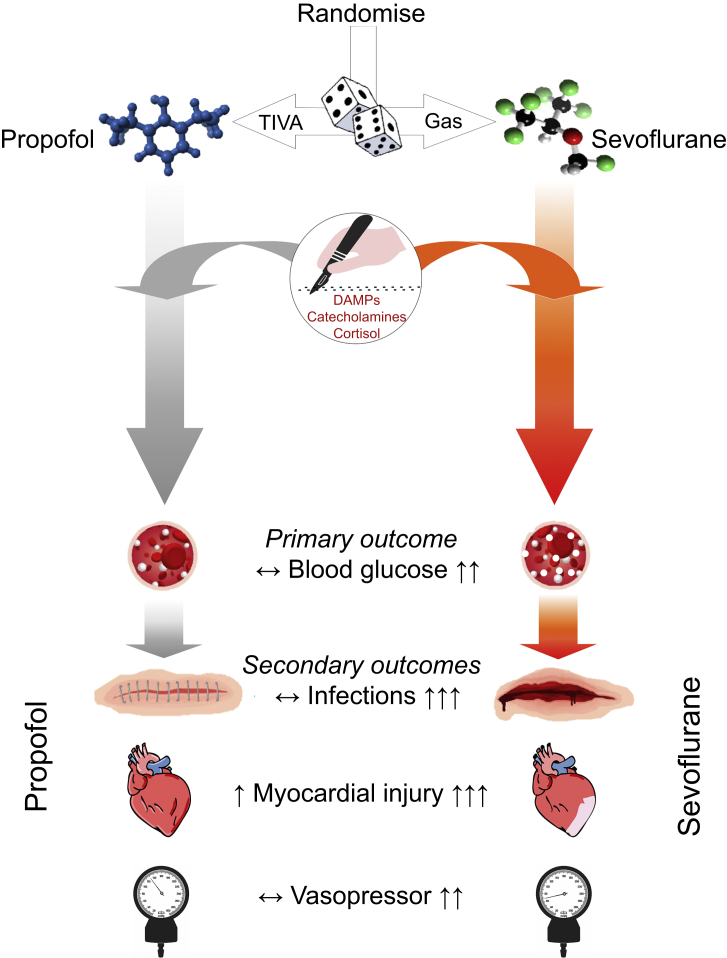
Hypothesised impact of mode of maintenance anaesthesia on blood glucose (primary outcome). Possible clinical secondary outcomes are also shown (postoperative infections, myocardial injury, vasoplegia). DAMP, Danger associated molecular patterns.
3. The interaction between mode of maintenance anaesthesia and glycaemic variability on infections after surgery.
4. The interaction between mode of maintenance anaesthesia and glycaemic variability on cardiovascular morbidity (myocardial injury, vasopressor use, major adverse cardiovascular events) after noncardiac surgery.
5. Preoperative metabolic endotypes (clinical and laboratory measures of insulin resistance), glycaemic control/variability, and complications after surgery.
6. Glycaemic variability and serial immuno-metabolic phenotypes.

This is an investigator-led study sponsored by the CI's substantive employer QMUL. The data collected will not be used to license/register any pharmaceuticals. Data arising from the research will be made available to the scientific community in a timely and responsible manner. An appointed lay representative will co-author a detailed scientific report, which will be submitted to a widely accessible scientific journal. Authorship of the final manuscript(s), interim publications, or abstracts will be decided according to active participation in the design, accrual of eligible participants, and statistical analysis. Contributing/participating investigators will be acknowledged in the final manuscript.

## Discussion

The GlucoVITAL trial will utilise real-time glucose monitoring by CGM to provide new insights into metabolic control in the perioperative period. The study design incorporates an assessment of the feasibility, and accuracy, of using CGM monitoring in the perioperative phase, plus exploratory measurements to further mechanistic understanding in the context of pragmatic clinical use. The setting of perioperative medicine is ideally suited to broaden the deployment of, and accessibility to, wearable metabolic technology such as CGM.^26^ In this way, re-examining the glycaemic stress response to major surgery may help improve perioperative outcomes by enhancing preparation for, and recovery from, major surgery in a personalised manner.^13^ The use of CGM data to quantify variability will enable us to confirm that absolute glucose concentrations are less mechanistically important than glucose variability, as high glycaemic variability is associated with higher mortality in critical care.^27^ The clinical outcome of non-critically ill, hospitalised patients having less glycaemic variability even with slight hyperglycaemia may be better than those having tight glycaemic control but higher glycaemic variability.^28^

## Funding

National Institute for Health and Care Research Health Technology Assessment (NIHR130573; VITAL RCT); National Institute for Health and Care Research/Medical Research Council Efficacy and Mechanism Evaluation Programme (NIHR 154842;GlucoVITAL study); National Institute for Health and Care Research Advanced Fellowship (NIHR300097 to GLA); British Heart Foundation (RG/19/5/34463 and RG/F/24/110146 to GLA); *British Journal of Anaesthesia*/Royal College of Anaesthetists (RCoA) Centenary investigator award (2024-27 to HJ); National Institute for Health and Care Research Biomedical Research Centre at The Royal Marsden NHS Trust and Institute of Cancer Research, London, UK (to SJ); Dexcom, Medtronic, and Roche Diabetes (to NSO).

## Conflict of interest

GLA is an editor of *British Journal of Anaesthesia. NSO* has participated in advisory groups for Dexcom, Medtronic, and Roche Diabetes and has received fees for speaking from Sanofi, Dexcom, Tandem, Medtronic, and Roche Diabetes. The other authors declare that they have no conflicts of interest.
